# Age and gender dependence of pre-contrast T1-relaxation times in normal human myocardium at 1.5T using ShMOLLI

**DOI:** 10.1186/1532-429X-14-S1-P221

**Published:** 2012-02-01

**Authors:** Stefan K Piechnik, Vanessa Ferreira, Adam J Lewandowski, Ntobeko Ntusi, Daniel Sado, Viviana Maestrini, Steven K White, Merzaka Lazdam, Rajarshi Banerjee, Mark B Hofman, James Moon, Stefan Neubauer, Paul Leeson, Matthew D Robson

**Affiliations:** 1 Department of Cardiovascular Medicine, University of Oxford, Oxford, UK; 2 Department of Physics & Medical Technology, ICaR-VU, VU University Medical Center, Amsterdam, Netherlands; 3 The Heart Hospital Imaging Centre, The Heart Hospital, London, UK

## Summary

Robust pre-contrast reference T1 values using ShMOLLI in 231 normal human controls aged 11 to 81 years demonstrate little dependence on gender, age or heart rates.

## Background

Quantitative T1-mapping is rapidly becoming a clinical Cardiovascular Magnetic Resonance (CMR) imaging tool that can distinguish normal from diseased myocardium. The usefulness of any quantitative measurement to identify disease lies in its ability to detect significant differences from an established normal range of values. In this study we aim to establish a large database for the normal range of T1 values in healthy human myocardium and to examine any differences based on age and gender.

## Methods

231 healthy volunteers underwent CMR with at least one ShMOLLI (Shortened Modified Look-Locker Inversion recovery) T1-map in one of three CMR centres. All data were acquired in 1.5T MR systems (Siemens, Avanto) using the ShMOLLI sequence for T1 maps as previously described [Piechnik at al. JCMR, 2010, 12:69] with 16 or 32 channel coil arrays. Each subject yielded a single average T1 value based on semi-automatically drawn myocardial contours in 3 short axis slices (typically, range 1-7, median=3, mean=3.3±1.1 slices). The heart rate (HR) was calculated from individual ShMOLLI image times.

## Results

**Figure 1:**
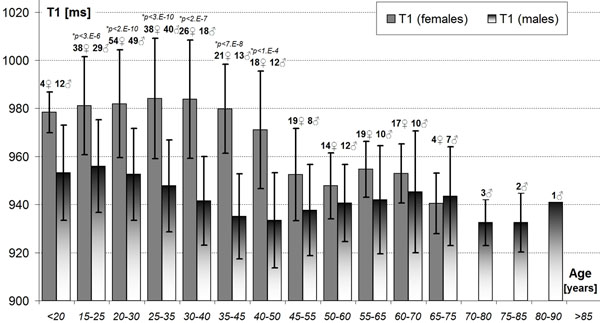
Gender and age dependence of T1±SD relaxation times in human myocardium at 1.5 Tesla. Note: Case numbers of females ♀ and males ♂ in the overlapping age groups are shown above the respective bars. (*)Unpaired Student T-test p-values for gender differences are marked when Bonferroni-significant.

The age range was 11-85 years with similar averages between females (37±14, 19-68 years old, n=123) and males (35±17, 11-85, n=108). The average T1 was 961±26ms amongst all subjects. Males had an average T1=947±20ms and females 974±25ms (p<0.0001). The gender differences in T1 are most prominent between the second and fifth decades of life (Fig. 1). T1 decreased with age at -0.5ms/year (females:-0.9ms/y; males: -0.3ms/y). There was only a very weak T1 dependence on HR (+0.45ms/(beat/minute), R=0.15, p=0.03), which was much smaller then previously reported +2.7ms/(b/m) for the MOLLI technique [Messroghli et al. Radiology 2006, 238:1004-1012]. Furthermore, there was no HR dependence for either gender separately and the overall relationship can be attributed to the concurrent differences in T1 and HR between groups in our material.

## Conclusions

Normal human myocardial T1 relaxation times can be measured precisely and show a narrow range of variation of about ±2% of the average in relation to age and gender but are not dependent on heart rate using ShMOLLI. T1 variability due to age and gender is small compared to the effect of major cardiac injuries, such as myocardial infarction which is characterised by 10-20% increase in T1. While normal variation will not impact on the sensitivity of T1-mapping to detect acute changes, for the detection of smaller T1 differences, age and gender matching between patients and controls may be desired.

## Funding

SKP, VMF, MDR funded by the NIHR Oxford Biomedical Research Centre Programme. VMF funded by the Alberta Heritage Foundation for Medical Research (AHFMR) and the University of Oxford Clarendon Fund Scholarship. DS and SW funded by British Heart Fundation.

